# Is TB Testing Associated With Increased Blood Interferon-Gamma Levels?

**DOI:** 10.3389/fvets.2017.00176

**Published:** 2017-10-23

**Authors:** Aideen E. Kennedy, Jim O’Mahony, Noel Byrne, John MacSharry, Riona G. Sayers

**Affiliations:** ^1^Animal and Bioscience Research Department, Animal and Grassland Research and Innovation Centre, Teagasc, Fermoy, Ireland; ^2^Department of Biological Sciences, Cork Institute of Technology, Cork, Ireland; ^3^Alimentary Pharmabiotic Centre, Biosciences Institute, University College Cork, Cork, Ireland

**Keywords:** Johne’s disease, TB test, interferon-gamma, purified protein derivative, ELISA

## Abstract

The Republic of Ireland reports a relatively low prevalence of Johne’s disease (JD) compared to international counterparts. Postulated reasons for this include a lower average herd size and a grass-based production system. Ireland also engages in high levels of bovine tuberculosis (bTB) testing. As interferon-gamma (IFN-γ) is believed to play a key role in protecting against JD, it is our hypothesis that administration of purified protein derivative (PPD), as part of the bTB test, is associated with a systemic increase in IFN-γ production, which may potentially limit clinical progression of the disease. We studied 265 cows (202 Friesian and 63 “Non-Friesian,” e.g., JerseyX, Norwegian Red) to assess IFN-γ levels and *Mycobacterium avium* subspecies *paratuberculosis* (MAP) antibody response before and after the bTB test. As part of the compulsory annual bTB test, avian and bovine PPD were administered at two separate cervical sites. To assess IFN-γ production, blood samples were taken before and 72 h after PPD administration. MAP antibody response was assessed before and 10 days post-PPD administration. A significant increase in MAP antibody response was identified post-bTB compared to pre-bTB response (*p* < 0.001). Additionally, IFN-γ production significantly increased at the post-bTB time point (*p* < 0.001) compared to the pre-bTB test readings. This may indicate a beneficial effect of bTB testing in controlling JD.

## Introduction

Mycobacteria are a leading cause of debilitating infections in domesticated animals and wildlife (1). Certain mycobacteria also have public health implications, such as bovine tuberculosis (bTB). Compulsory eradication schemes were initiated in the Republic of Ireland in the 1960s to both protect human health and agricultural exports (2). Although levels of TB in the human population in Ireland have dramatically reduced in the past 50 years, bTB eradication remains necessary in order to comply with European trading conditions (Directive 64/432 EEC) (2).

Bovine tuberculosis is caused by *Mycobacterium bovis* (*M. bovis*). In the late 1800s, Robert Koch identified the tubercle bacillus and also developed tuberculin, a glycerol extract of pure culture of tubercle bacilli (3). The intradermal skin test has been used diagnostically for detection of human and bovine TB for over 100 years. Originally performed using Koch’s Old tuberculin, a more stable and consistent preparation known as purified protein derivative (PPD), is now used for intradermal tests. PPD is produced by growing a mycobacterial organism on liquid culture, subsequently heat treating, filtering, washing, and then re-dissolving into a sterile preparation free from intact mycobacteria (4, 5). When injected intradermally, PPD provides an antigen source to identify animals whose immune system has been sensitized by previous exposure to the mycobacterium. Sensitization in an animal is identified by development of an edematous lesion at the site of injection.

Antemortem screening for bTB in Ireland is conducted using the single intradermal cervical comparative test (SICCT). All bovines over 6 weeks of age are tested a minimum of once per year. Good et al. (6), on analyzing results from 1,703 herds restricted with bTB, found over 18% of animals (10,962) received two tests with a small number of animals (7) tested five times in a single year. While many countries use the single intradermal test, where *M. bovis* PPD is administered in isolation, the SICCT utilizes intradermal introduction of both *M. bovis* (bPPD) and *M. avium* (aPPD) PPD at two different sites on the neck (5). A relative difference in the size of the edematous lesion of >4 mm at the bPPD injection site compared to aPDD site indicates a positive result (4).

Interferon gamma (IFN-γ) is an inflammatory cytokine that is critical to both innate and adaptive immune systems (7) across mammalian species. It is an important activator of macrophages following bacterial exposure (8). IFN-γ in more recent decades has been applied as a tool in the diagnosis of mycobacterial diseases such as bTB and human tuberculosis (5, 9), Johne’s disease (JD) (10), and leprosy (11).

The interferon-gamma assay is approved by the EU for bTB testing (5) and is used as an ancillary test in the Irish bTB eradication scheme. The IFN-γ test involves incubation of heparinized blood samples in the presence of test antigens (avian and bovine PPD). Subsequently, plasma supernatant from each blood aliquot is harvested. IFN-γ production is estimated using an enzyme immunoassay (EIA) (12). IFN-γ testing can either be used to confirm bTB SICCT positive reactors or alongside intradermal tests to increase diagnostic sensitivity (13).

Purified protein derivative administration has been shown to boost *in vitro* IFN-γ production in the case of bTB (14, 15). Ota et al. (16) found skin testing alone significantly induced PPD-specific IFN-γ producing cells in humans. Furthermore Thom et al. (17) conducted a study to determine the effect of repeated skin testing on immune responses of calves following experimental infection with *M. bovis*. Prior to experimental infection, during an undefined transient infection (as evidence by an IL10 response), the authors noticed that, compared to calves that received multiple skin tests, control animals appeared to have higher proliferative responses and IFN-γ synthesis. The authors proposed that multiple skin tests had either led to a suppressed response to the transient infection or alternatively that skin testing had aided protection against this transient infection, thus highlighting important interactions between PPD administration and host immune responses.

An additional pathogenic and speculated zoonotic member of the Mycobacteriaceae is *Mycobacterium avium* subspecies *paratuberculosis* (MAP), the causative agent of JD in cattle (18). Currently, there is no effective treatment for JD, and control is based on breaking the cycle of transmission to susceptible animals by limiting contact with MAP-infected feces, colostrums, and milk (19). It is generally believed that early subclinical MAP infections result in a cell-mediated immune response involving delayed type hypersensitivity (DTH) with production of cytokines by T-lymphocytes, including IFN-γ (20, 21). Indeed, IFN-γ has been established as an important cytokine in host defenses against JD (21).

Ireland reports a relatively low prevalence of MAP sero-positivity (22). Additionally, Kennedy et al. (23) have hypothesized that Irish cows may be less susceptible than international counterparts to developing clinical signs (progressive emaciation and diarrhea) of JD. Reasons suggested for this include low average herd size (75 cows), a predominantly grass-based diet, and the comprehensive bTB testing regime conducted in Ireland (23, 24). In terms of investigating this further, we sought to examine the impact of administration of avian and bovine PPD, as part of the required annual SICCT test, on *in vivo* levels of plasma IFN-γ. Our overall objective was to elucidate whether bTB testing in Ireland could be contributing to MAP control/suppression in Irish herds.

## Materials and Methods

### Study Cows

A total of 265 cows were recruited to the study in April 2016 from a farm that had been depopulated in 1997 following confirmation of a case of BSE. The current herd, therefore, consisted of descendants of cows used to repopulate the farm (25). Since establishment of the new herd in 2000, cows from 47 different herds of origin had been purchased into the herd. Breed, age, parity, MAP ELISA status, and bTB status was available for each cow. Approximately 85% of the herd were spring-calving (i.e., calving between 1st January and 30th April). Herd SICCT history was examined in advance of recruitment of the herd to the study and revealed minimal issues with bTB (no reactors in 5 years). All, bar eight animals in the current study, were from the same herd of origin and, therefore, were subjected to similar frequency of annual SICCT. All animals had previously partaken in a minimum of two SICCTs. The herd had been enrolled in the voluntary Animal Health Ireland (AHI) pilot JD control programme since 2014. All animals were examined for clinical signs of JD prior to study initiation. Although the study herd contained six MAP ELISA positive animals, no animal displayed clinical signs of JD.

### Single Intradermal Cervical Comparative Test

The routine annual SICCT was administered by the farm’s private veterinary practitioner in May 2016. It was conducted in line with standard Department of Agriculture guidelines (26). Briefly, on the middle one third of the neck, two injection sites (dorsal and ventral), 12.5 cm apart, were clipped and skin-thickness measurements recorded with calipers. McLintock syringes (Duggan Veterinary, Ireland) were used to administer 0.1 mL avian PPD dorsally and 0.1 mL of bovine PPD at the ventral site. PPD was supplied by DAFM in line with statutory requirements. Seventy-two hours post-PPD administration skin thickness measurements at both injection sites were re-assessed to evaluate the presence or absence of a DTH response.

### Blood Samples

Blood samples were collected pre- and 72 h post-PPD administration. Pre-SICCT samples were tested for both IFN-γ and MAP serological response. The 72-h post samples were tested for IFN-γ levels only. This time-point was chosen as Coad et al. (27), reported a statistically significant increase in *in vitro* IFN-γ response 3 days post-bPPD administration. Based on results by Kennedy et al. (24) and Kennedy et al. (23) indicating increased production of MAP antibodies post-PPD administration, a blood sample was collected ten days post-SICCT to examine the serological response on MAP ELISA. The sampling protocol was approved by the Teagasc Animal Ethics committee and a study license was granted by the Irish Health Products Regulatory Authority (HPRA). Blood samples were taken from the coccygeal vein using 20-Gauge needles into evacuated lithium heparin blood sampling tubes. Samples were centrifuged for 15 min at 3,000 *g* and plasma aspirated for same-day testing.

### MAP ELISA

*Mycobacterium avium* subspecies paratuberculosis ELISA tests were conducted by a commercial ISO17025 accredited laboratory (Enfer Kildare), designated by AHI for the Irish voluntary JD control programme. Samples were tested using the ID Screen Paratuberculosis Indirect Screening Test (ID Vet, Montpellier, France). Results were reported as sample to positive ratios (S/P ratio) calculated using the formula S/P ratio = [(OD Sample − OD Negative control) ÷ (OD Positive control − OD Negative control) × 100]. The test is an *M. phlei* absorbed ELISA, which detects anti-MAP IgG. For the purposes of reporting within-herd MAP prevalence, ELISA S/P ratio results were categorized according to manufacture instructions, i.e., samples recording S/P ratios of ≥70 S/P categorized as seropositive.

### IFN-γ Sample Preparation and Testing

Samples for IFN-γ were tested using a modified version of Bovigam IFN-γ kit (Celtic Diagnostics Ltd., Dublin 22, Ireland). The first step in this test typically involves a blood culturing step, involving addition of a negative control antigen, avian PPD, and bovine PPD to three separate aliquots of whole blood and incubated overnight. The second stage comprises measuring the production of IFN-γ (absorbance value) from the stimulated lymphocytes in separated plasma using a monoclonal antibody-based sandwich EIA. For our purposes, we used the intradermal administration of PPD to study cows as the lymphocyte stimulation step, and following centrifugation, assayed plasma directly using the IFN-γ EIA. This was deemed a suitable methodology as our purpose was to compare *in vivo* IFN-γ levels pre- and post-PPD administration as opposed to identifying bTB infected cattle. All plasma samples for IFN-γ were plated within 6 h of blood collection from cows.

Samples were assayed in duplicate and results reported as optical density values at 450 nm (OD_450_). The conventional kit interpretation is calculated by comparing mean negative control antigen, avian PPD, and bovine PPD OD_450_ values. In the current study, we simply compared mean pre- and post-SICCT plasma IFN-γ levels as all cows were required to be administered with both avian PPD and bovine PPD. It should be noted that two ELISA plates recorded negative controls outside manufacturer recommendations (Recommended Mean Negative Control < 0.13. The two plates recorded negative controls of 0.15 and 0.186, respectively). As the kit was not being used as conventionally specified, it was decided to perform analysis including and excluding results from both plates (*n* = 264 vs. *n* = 179 cows). Analysis yielded broadly similar results, analysis relating to breed differences, however, changed to recording a tendency (*p* = 0.088) rather than statistical significance (*p* = 0.04). Results reported are from *n* = 264 cows. In the interest of completeness, values (maximum, median etc.) from *n* = 179 cows are provided throughout the manuscript and labeled “analysis 179 cows.” Additionally, figures and tables from “analysis 179 cows” are provided as supplementary files.

### Cow Classification

For SICCT, all cows were classified based on relative differences in skin thickness measurements at avian and bovine PDD injection sites. An animal displaying a >4 mm skin thickness increase at bovine compared to avian injection sites was classified as a positive reactor, an increase of >2–4 mm as an inconclusive, and animals with lower or no increases in skin thickness measurements classified as negatives. In terms of MAP ELISA, manufacturer positive cut-off values were applied to classify cows as seropositive or seronegative. Cows were also classified on the basis of breed (Holstein-Friesian, non-Holstein-Friesian) and parity (1–8).

### Data Analysis

Descriptive analysis and dataset construction were completed in Excel (MS Office 2010). Figures were constructed using Excel (MS Office 2010) and GraphPad Prism. Normality of continuous datasets was examined visually using ladders of power histograms. Statistical analyses were completed using Stata version 12 (StataCORP, USA). Wilcoxon rank sum tests were used to investigate differences between pre- and post-SICCT IFN-γ OD_450_ results, pre- and post-SICCT MAP ELISA results, avian PPD site measurements, and bovine PPD site measurements (continuous variables). Univariable linear regression was initially used to examine the association between IFN-γ production (dependent variable) and sampling time point (pre-SICCT vs. post-SICCT), breed (Friesian vs. non-Friesian), parity (1, 2, 3, 4, 5, 6, 7, 8), changes in skin thickness measurement due to DTH pre- and post-avian PPD administration (continuous variable in millimeter) and skin thickness measurement due to DTH pre- and post-bovine PPD administration (continuous variable in millimeter). Difference between pre and post-SICCT MAP ELISA S/P ratio was also examined as an independent variable. A multivariable generalized estimating equation (GEE) was subsequently built using independent variables, which yielded *p*-values ≤0.10 (Table 1) in univariable models.

**Table 1:** Univariable analysis examining associations between variables.

	Breed	Parity	Pre-single intradermal cervical comparative test (SICCT) IFN	Post-SICCT IFN	Pre-SICCT *Mycobacterium avium* subspecies paratuberculosis (MAP)	Post-SICCT Map	Avian response	Bovine response	Difference IFN pre/post SICCT
Parity	0.77								
Pre-SICCT IFN	0.76	0.23							
Post-SICCT IFN	**0.041**	0.84	0.63						
Pre-SICCT MAP	0.41	**0.02**	0.12	0.31					
Post-SICCT Map	0.21	0.96	**0.05**	0.77	**<0.001**				
Avian response (mm)	0.81	0.69	0.31	0.8	**0.04**	**<0.001**			
Bovine response (mm)	0.58	0.39	0.28	**0.08**	**0.04**	**<0.001**	**<0.001**		
Difference IFN Pre/post SICCT	**0.06**	0.64	**<0.001**	**<0.001**	0.53	0.88	0.63	**0.05**	
Difference MAP response Pre/post SICCT	0.29	0.39	**0.06**	0.89	0.12	**<0.001**	**<0.001**	**<0.001**	0.58

*p-Values in bold highlight variables subsequently included in logistic regression models*.

A second GEE analysis was conducted to investigate associations between MAP ELISA S/P ratio (dependent variable) and the independent variables described previously, namely, sampling time point, breed, parity, and skin thickness measurements at avian and bovine PPD injection sites. Difference between pre and post-SICCT IFN-γ production was also examined as an independent variable. GEE models were constructed by backwards elimination with a forward step. Second level interactions between independent variables were examined and included in both models at *p* ≤ 0.05. A Gaussian distribution and an identity link function were used.

## Results

### Herd Information

The study herd consisted of 202 Friesians or Friesian crosses and the remainder were predominantly Jersey or Jersey crosses and a small number of Norwegian red (21) (Analysis 179 cows: 138 Friesians, 26 Jersey, 15 Norwegian red).

### Single Intradermal Cervical Comparative Test

A total of two animals were classified as inconclusive on SICCT but none were classified as bTB positive (inconclusive 1: skin measurements at bPPD site increased from 7 to 10 mm, no increase in skin thickness was recorded at aPPD. The second inconclusive animal recorded an increase of 7 mm at the bPPD site and 3 mm at the aPPD site: Both inconclusive animals were subjected to repeat SICCT at a 60-day interval and tested bTB negative). In all, 91 animals exhibited DTH to aPPD and the maximum increase in skin thickness recorded at the avian PPD site post-SICCT was 11 mm (range 0–11 mm, median 0 mm). With regard to bovine PDD, 36 animals recorded DTH against it (range 0–7 mm, median 0 mm), 35 of whom had also recorded avian PPD DTH [analysis 179 cows: 61 animals exhibited DTH to aPPD (range 0–11 mm, median 0 mm), 20 animals recorded DTH to bovine PDD (range 0–4 mm, median 0 mm), 19 of whom had also recorded avian PPD DTH].

### IFN-γ Plasma Detection

**Figure 1:**
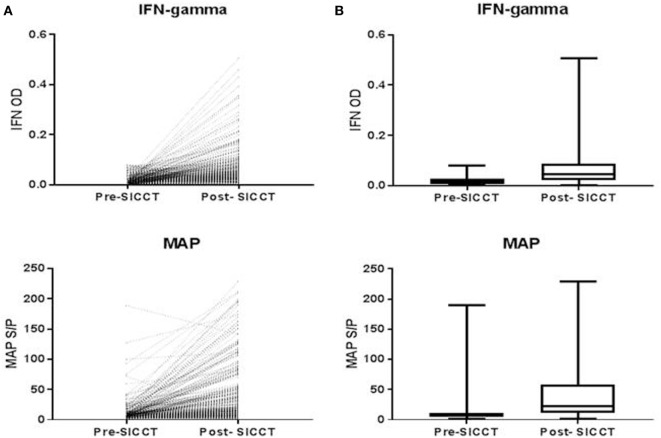
**(A)** Shows individual interferon-gamma (IFN-γ) and *Mycobacterium avium* subspecies paratuberculosis (MAP) value recorded for each cow both pre and post-single intradermal cervical comparative test (SICCT). **(B)** Shows Spear style box plots of IFN-γ and MAP ELISA response both pre- and post-SICCT (*n* = 264). Wilcoxon rank sum identified significant differences between pre- and post-SICCT IFN-γ OD450 results and pre- and post-SICCT MAP ELISA results.

The pre-SICCT IFN-γ OD450 ranged from 0.001 to 0.08 (median 0.02) [analysis 179 cows: 0.001 to 0.08 (median 0.02)]. Post-SICCT IFN-γ measurements ranged from 0.001 to 0.507 (median: 0.05) (Figure 1) [analysis 179 cows: range 0.001–0.507 (median: 0.03)]. A single cow recorded a pre-SICCT IFN-γ measurement of 0.7405 and was, therefore, removed from the analysis as an outlier; this animal showed no increase in skin thickness at either the aPPD or bPPD injection site and post-PPD IFN-γ response was in line with the remainder of the herd (0.25). Statistically, post-SICCT IFN-γ measurements were significantly higher than pre-SICCT OD_450_ values (*p* < 0.001) (analysis 179 cows: p < 0.001). Similarly, the multivariable GEE analysis highlighted a statistically significant increase in IFN-γ production post-SICCT (Table 2). It should be noted, however, that 62 animals recorded either no change or a decrease in IFN-γ response. A lower increase in IFN-γ production post-SICCT was recorded in non-Holstein-Friesian compared to Holstein-Friesian cows (*p* = 0.04) (Table 2) (analysis 179 cows: *p* = 0.088).

**Table 2:** Significant associations between interferon-gamma (IFN-γ) production and independent variables (*n* = 264).

Dependent variable	Coefficient	*p-*Value	95% conf. interval	Model (model *p-*Value)
Independent variable					
**IFN-γ production**				
Post-single intradermal cervical comparative test (SICCT) vs. pre-SICCT	0.05	<0.001	0.04, 0.06	testing time point, breed, (*p*: < 0.001)
Non-Friesian vs. Friesians	−0.01	0.04	−0.02, −0.001	

### MAP ELISA

Prior to the administration of avian and bovine PPD, six animals were classified as MAP ELISA positive with S/P ratios ranging from 72 to 189 (median = 97.1). Post-SICCT, 64 animals were classified as MAP ELISA positive, values ranged from 70 to 229 with a median S/P ratio of 112.6 [analysis 179 cows: pre-SICCT four animals were classified as MAP ELISA positive with S/P ratios ranging from 75.7 to 189 (median = 113.89). Post-SICCT, 43 animals were classified as MAP ELISA positive, values ranged from 75 to 229 with a median S/P ratio of 112].

**Figure 2:**
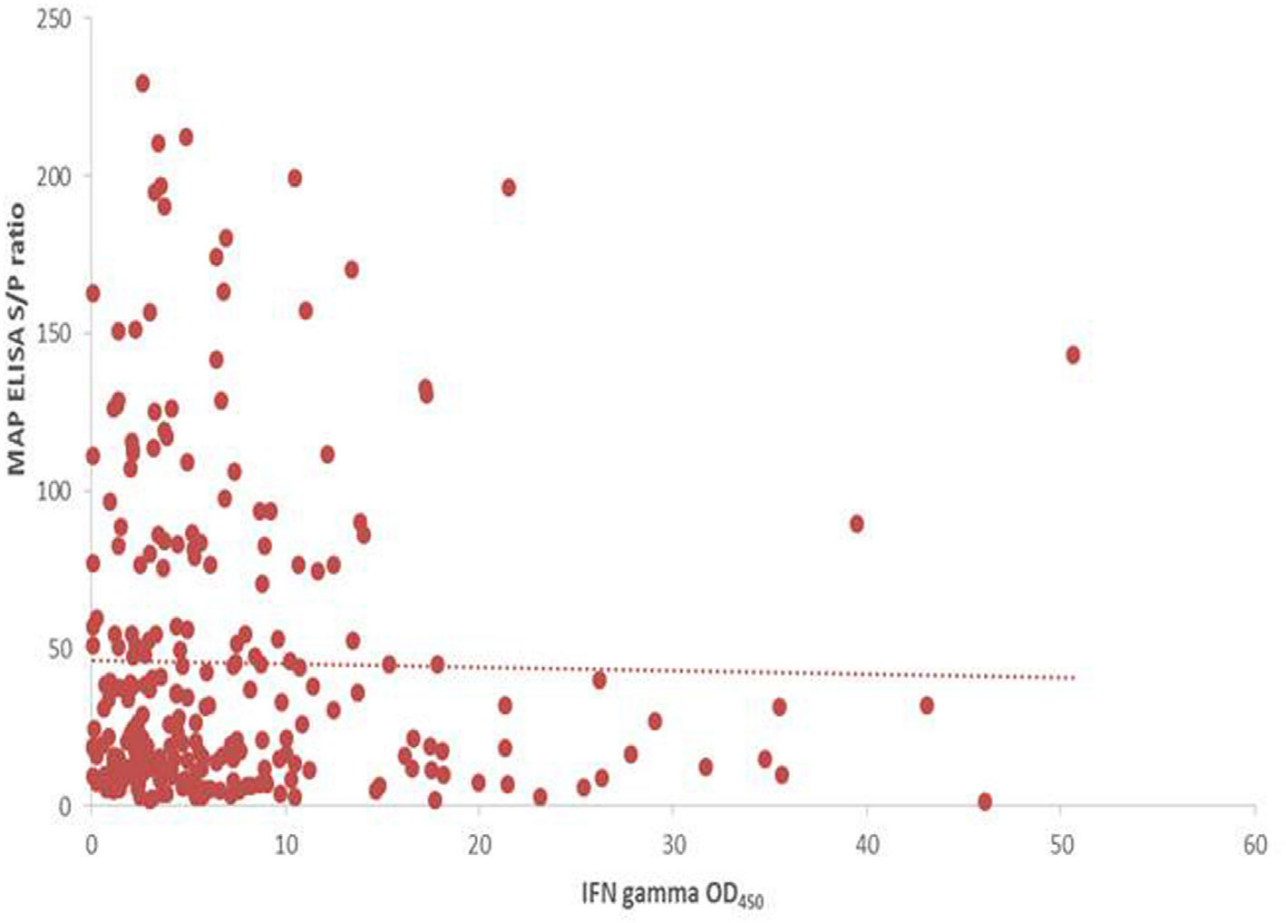
Scatter plot showing the relationship between post SICCT interferon-gamma (IFN-γ) and MAP ELISA antibody response (*n* = 264). IFN-γ × 1,000 to aid visualization.

A significant increase in MAP serological response was identified post-SICCT compared to pre-SICCT response (*p* < 0.001) (Figure 2) (Analysis 179 cows: *p* < 0.001). A significant association was identified between MAP S/P ratio and both avian and bovine PPD DTH response. For every 1 mm increase in bovine PPD DTH, an increase in MAP S/P ratio of 4.4 was identified (*p* = 0.013). Similarly for every 1 mm increase in avian DTH, an increase in MAP S/P ratio of 3.4 was recorded (*p* < 0.001) (Table 3). Univariable linear regression identified no association between post-SICCT IFN-γ and antibody production. A scatter plot in Figure 2 shows the relationship between each variable.

**Table 3:** Significant associations between *Mycobacterium avium* subspecies paratuberculosis (MAP) ELISA response and independent variables (*n* = 264).

Dependent variable	Coefficient	*p-*Value	95% conf. interval	Model (model *p-*Value)
Independent variable				
**MAP ELISA response**				
Post-SICCT vs. pre-SICCT	33.9	<0.001	28.5, 39.4	Testing time point, Avian purified protein derivative (PPD) delayed type hypersensitivity (DTH) response, Bovine PPD DTH response (*p-*Value: <0.001)
Avian PPD DTH response (mm)	3.4	<0.001	1.7, 5.0	
Bovine PPD DTH response (mm)	4.4	0.013	0.1, 7.9	

## Discussion

The primary aim of this study was to investigate if the administration of PPD during the routine annual SICCT for bTB was associated with an increase in circulating plasma IFN-γ. The significant association highlighted between PPD administration and an increase in systemic IFN-γ provides support for the theory that SICCT may have a potential immuno-protective influence in Irish cattle with regard to MAP infections and may contribute to the relatively low levels of overt clinical signs of JD experienced on Irish farms (24).

Interferon-gamma, originally called macrophage-activating factor (28), orchestrates a diverse array of cellular activities, including upregulation of pathogen recognition, antigen processing, and antigen presentation. During infection, IFN-γ-induced actions result in heightened immune system function and surveillance, with IFN-γ amplifying immune system response to pathogens (28). IFN-γ has been established as an important cytokine in defenses against mycobacterial disease (21, 29, 30) and appears important in limiting infection by MAP (31). In human medicine, IFN-γ has been used as a therapeutic adjuvant in wide range of diseases from atopic dermatitis (32) to ovarian cancer (33). Marciano et al. (34) found that prophylactic use of IFN-γ was effective in patients with chronic granulomatous disease. Given the role of IFN-γ in limiting progression of MAP infections (31) and its use as a therapeutic adjuvant and prophylactic agent, the increased level of IFN-γ recorded post-PPD administration in the current study may indicate TB-testing can contribute to MAP control in Irish dairy cows. As some animals in Ireland face up to five tests in 1 year (6), the suggested protective effect of TB-testing may help limit progression of MAP infections.

Conflicting reports exist in the literature relating to PPD administration stimulating increased production of IFN-γ *in vitro*. Indeed, in the current study, 62 animals did not record an increase in IFN-γ. Many studies report no significant effect observed after PPD administration (27, 35, 36), while others (14, 15) found increased IFN-γ responses. On reviewing relevant literature, Schiller et al. (37) found it could not be disregarded that skin testing induces an IFN response particularly in animals sensitized to environmental mycobacteria. It has been reported by Buddle et al. (38) that many animals are naturally sensitized to environmental mycobacteria at a young age and develop an immunological response to such antigens by 6 weeks of age. Ireland is recognized as having an abundance of environmental mycobacteria (39). As Irish livestock systems are largely grass-based with animals only housed for 2–3 months per year, potential exposure to such mycobacteria is probable. It is possible; therefore, that prior infection with environmental mycobacteria is contributing to the significant increase in the IFN-γ response post-SICCT recorded in our study. Hope et al. (40) also reported prior exposure to *M. avium* induces low level protection to *M. bovis* and may prime host immune responses. As to whether this could be extrapolated to environmental mycobacteria inducing low level protection against MAP in Irish cattle requires further investigation, but deserves serious consideration.

Vaccines have been available for JD since the early 20th century. The majority of vaccines are based on killed mycobacterium cells in an oil-based adjuvant. Use is prohibited in a number of countries due to interference with TB diagnostics (41). If results in the current study could be expanded upon and it was shown that PPD was exerting a vaccine-like effect, it may highlight a new control approach for areas not currently engaging in bTB testing programmes to a similar extent as Ireland. Although, not perfect as clinical JD, does occur in Ireland, few vaccines will report 100% efficacy (41). Research is required to see if a modified PPD preparation and administration protocol could provide a comparable response to disease as provided by commercial vaccines.

An interesting finding from this study is that non-Holstein-Friesian breeds, consisting predominantly of Jersey and Jersey cross cows, produced less IFN-γ in the post-PPD period than Holstein-Friesian and crosses. A number of studies have reported an increased likelihood of Channel Island breeds testing MAP positive (42–44). Verschoor et al. (45) reported differing expression of genes related to immune response and antigen processing in MAP infected Friesians versus Jerseys. Ballou (46) demonstrated reduced immune responses to *E-coli* infection in Jersey calves compared to Holstein-Friesians. More specifically, of four MAP fecal culture positive animals detected in a single Irish mixed-breed herd over a 5-year period, all were of Jersey ancestry (Kennedy et al., unpublished data). It is possible, therefore, that Channel Island cow breeds have a lesser ability to mount an immune response effective at clearing MAP infection. Indeed, genomic variation within the IFN-γ gene may account for differences between breeds (47). From a purely practical point of view, potentially more stringent MAP-related management practices should be considered in herds containing Channel Island breeds to minimize JD transmission. Such an approach could also include a more severe interpretation of ELISA results during MAP herd surveillance. As the heritability of MAP susceptibility in Jersey cows has been reported at between 8 and 27% (48), an appropriate breeding programme could also greatly assist in improving MAP outcomes in Jersey cows.

In agreement with previous studies (24, 49), administration of PPD was associated with an increase in MAP antibody response. The current study only examined the response at day 10 post-PPD administration, but previous research on Irish dairy herds (24) has recommended avoiding testing for MAP antibodies until day 71 post PPD to avoid potential interference with MAP ELISA results. An association was likewise identified in this study between DTH skin reactions and MAP ELISA antibody response in agreement with a previous study (50). In that study, it couldn’t be determined whether increases in post-PPD MAP antibody responses were due to increased sensitivity at detecting MAP-infected animals, or as a result of decreased specificity due to cross reacting antibodies. Interestingly, Hostetter et al. (51) reported that given the appropriate environment, opsonization of MAP with specific antibodies can lead to an oxidative burst and reduced survival of MAP. In the study by Hostetter et al. (51), MAP bacteria were opsonized for 24 h with serum from animals confirmed positive for MAP antibodies. According to Kennedy et al. (24), a statistically significant increase in MAP antibody response persists until 71 days post-PPD administration, indicating the post-PPD increase in MAP antibody response would exist for a sufficient time to allow opsonization of bacteria to occur. Potentially, this may indicate that MAP antibodies produced post the administration of SICCT, may contribute to the control of MAP. Further research would be required, however, as other researchers have reported the ineffectiveness of antibodies in controlling MAP.

Although formal comparison of MAP prevalence across studies is difficult due to variation in study design (52), the herd prevalence reported in Ireland (21.4% of herds reporting at least one ELISA positive) (22) is relatively low, compared to a number of international counterparts. A review of MAP prevalence across Europe reported a between herd prevalence “guesstimate” of >50% (52). Different bTB testing regimes operate internationally. Many countries do not implement a minimum of a once annual SICCT as occurs in Ireland. In the majority of European countries that engage in bTB testing, bovine PPD is applied to the cervical region (cervical single intradermal test; SIT) (53). Indeed, a number of European countries are officially bTB free and don’t conduct routine testing for bTB, including the Netherlands (54), who report a MAP prevalence of between 31 and 71% (55). Elsewhere, in North America and the Southern Hemisphere, bovine PPD is applied *via* the caudal fold test. In these regions, MAP prevalence also appears greater than Ireland. A herd level apparent prevalence of 70.4% was reported in US dairy herds by Lombard et al. (56). Vilar et al. (57) have reported prevalence’s of 26.6–41.4% in certain regions of Brazil. In Ireland and the UK, poor specificity is reported for the SIT; therefore, the SICCT is used to test for bTB (53). Although the SICCT is used in both countries, differences exist in the frequency of bTB testing. Within the UK testing intervals of up to 4 years are permitted in certain regions. Perhaps, the reduced frequency of bTB testing conducted in the UK compared to Ireland accounts for the increased prevalence of MAP reported in the UK [68% reported by Velasova et al. (58)]. It would be interesting to study whether areas engaging in bTB testing every 4 years report higher MAP prevalence compared to those areas within the UK testing annually.

It should be noted that the relatively low prevalence of MAP in Irish herds may be attributable to a number of other factors. Previously suggested reasons include outdoor grazing systems and a lower average herd size compared to international counterparts (23). It has also previously been suggested that TB testing may contribute to JD control, as MAP-infected animals can show false positive reactions to SICCT and be removed from the herd. This study is the first of our knowledge to hypothesize if TB-testing may contribute to MAP control in Ireland and investigate if PPD can induce production of cytokines known to be immunologically important in the control of JD. Results are suggestive, however, that bTB-testing may be contributing to JD control and requires comprehensive investigation. This current study, however, involves a single herd only at one bTB test only and more comprehensive studies are required to further investigate our hypothesis. In addition to inclusion of an increased number of herds, future work should also examine T-cell subsets and include investigation of mRNA expression levels. Examining the length of time, the IFN-γ response persists would also be valuable. Equally Stabel et al. (59), when examining IFN-γ response post intradermal stimulation with johnin found greater IFN-γ production in subclinically infected animals compared to control and clinically infected animals. Therefore, in future studies, it would be interesting to include herds with both clinically and subclinically infected cows and examine their respective post-PPD IFN-γ responses.

Although representing different arms of the immune response, the association between post-SICCT IFN-γ and antibody production was examined. While there was an increase in both post-SICCT IFN-γ and antibody production, no association was highlighted between them. As Mikkelsen et al. (60) reports that cell-mediated responses can control or eradicate MAP, it may indicate that the animals with increased IFN-γ production in the current study are limiting the progression of the disease, and not producing significant IgG antibody levels. Indeed, studies demonstrate that IFN-γ enhances macrophage autophagy function (61). The increased plasma IFN-γ levels may suggest enhanced macrophage autophagy and clearance of mycobacteria. Surprisingly, no association was identified between systemic IFN-γ production and DTH responses. Over 15 different cytokines, however, have been identified at the local site of tissue inflammation (62). In humans, it is known that TNF-α is more effective in inducing a local DTH response (62, 63). As a number of cytokines are involved in the DTH reaction, not just IFN-γ, it may explain the lack of association between IFN-γ production and DTH responses.

## Conclusion

We can conclude from this study that administration of PPD as part of the bTB test is associated with an increase in MAP antibody response. Associations were also identified between skin DTH responses to the bTB test and post-bTB test MAP antibody response. Additionally, IFN-γ production was significantly increased at the post-bTB time point. The immune response identified post-bTB test administration may indicate a role for TB testing in controlling JD.

## Ethics Statement

The study documented in this manuscript was approved by the Irish Health Products Regulatory Authority (HPRA). The Teagasc Animal Ethics Committee approved the study prior to license application with the HPRA.

## Author Contributions

AK was involved in conception and design of the work, sample collection, data analysis, and interpretation of data for the paper. AK drafted the initial manuscript and revised it critically and gave final approval of the version to be published. JM was involved in conception and design of the work, analysis and interpretation of data for the work. JM critically revised the work and gave final approval of the version to be published. NB was involved in conception and design of the work, sample collection, and interpretation of data for the work. NB critically revised the work and gave final approval of the version to be published. JS was involved in conception and design of the work, analysis, and interpretation of data for the work. JS critically revised the work and gave final approval of the version to be published. RS was involved in conception and design of the work, data analysis, and interpretation of data for the paper. RS was involved in drafting the initial manuscript and revised it critically and gave final approval of the version to be published.

## Conflict of Interest Statement

The authors declare that the research was conducted in the absence of any commercial or financial relationships that could be construed as a potential conflict of interest.
