# Supplementary material for: Is TB Testing Associated With Increased Blood Interferon-Gamma Levels?
Source: Front Vet Sci. 2017 Oct 23;4:176. doi: 10.3389/fvets.2017.00176 (PMC5660059; doi:10.3389/fvets.2017.00176)
Supplement: Supplementary file 1 [file Table_1.PDF]

Supplementary Material

Is TB testing lowering the risk of clinical Johne's disease in the Republic of Ireland?

Aideen E. Kennedy[1,2], Jim O'Mahony[2], Noel Byrne[1], John Mac Sharry[3], Riona G. Sayers[1*]

*Corresponding Author: riona.sayers@teagasc.ie

Supplementary Table 1: Univariable analysis examining associations between variables. P-values in bold highlight variables subsequently included in logistic regression models (n=179).

	**Breed**	**Parity**	**Pre-SICCT IFN**	**Post-SICCT IFN**	**Pre-SICCT MAP**	**Post-SICCT MAP**	**Avian response**	**Bovine response**	**Difference IFN Pre/Post SICCT**
**Parity**	0.99								
**Pre-SICCT IFN**	0.75	0.74							
**Post-SICCT IFN**	**0.07**	0.62	0.56						
**Pre-SICCT MAP**	0.23	0.12	0.23	0.83					
**Post-SICCT Map**	0.25	0.92	0.20	0.63	**<0.001**				
**Avian response (mm)**	0.95	0.42	0.41	0.78	**0.09**	**<0.001**			
**Bovine response (mm)**	0.79	0.56	**0.09**	0.32	**0.002**	**<0.001**	**<0.001**		
**Difference IFN Pre/Post**	**0.07**	0.70	**<0.001**	**<0.001**	0.93	0.43	0.90	0.17	
**Difference MAP Pre/Post**	0.46	0.40	0.36	0.51	0.22	**<0.001**	**<0.001**	**<0.001**	0.39

Supplementary Table 2: Associations between IFN-γ production and independent variables (n=179)

**Dependent Variable**<br>Independent Variable	**Coefficient**	***P* Value**	**95% Conf. Interval**	**Model** (Model *P* value)
**IFN-γ Production**				
Post-SICCT vs. Pre- SICCT	0.03	<0.001	0.02, 0.04	Testing Time point, Breed, (*P:* <0.001)
Non- Friesian vs. Friesians	-0.01	0.088	-0.02, 0.001	

Supplementary Table 3: Significant associations between MAP ELISA response and independent variables (n=179)

**Dependent Variable**<br>Independent Variable	**Coefficient**	***P* Value**	**95% Conf. Interval**	**Model** (Model *P* value)
**MAP ELISA Response**				
Post-SICCT vs. Pre- SICCT	33.0	<0.001	26.8, 39.3	Testing Time point, Avian PPD DTH response, Bovine PPD DTH response
Avian PPD DTH response (millimetres)	3.9	<0.001	2.0, 5.8	(*P* value: <0.001)
Bovine PPD DTH response (millimetres)	10.0	0.001	4.3, 15.7	

Supplementary Figure 1: Column A shows individual IFN-γ and MAP value recorded for each cow both pre and post-SICCT. Column B shows box plots of IFN-γ and MAP ELISA response both pre- and post-SICCT (n=179)

Supplementary Figure 2: Scatter plot showing the relationship between post SICCT IFN-γ and MAP ELISA antibody response (n=179). IFN-γ *1000 to aid visualization.
