# Supplementary figures and images for: Is TB Testing Associated With Increased Blood Interferon-Gamma Levels?
Source: Front Vet Sci. 2017 Oct 23;4:176. doi: 10.3389/fvets.2017.00176 (PMC5660059; doi:10.3389/fvets.2017.00176)

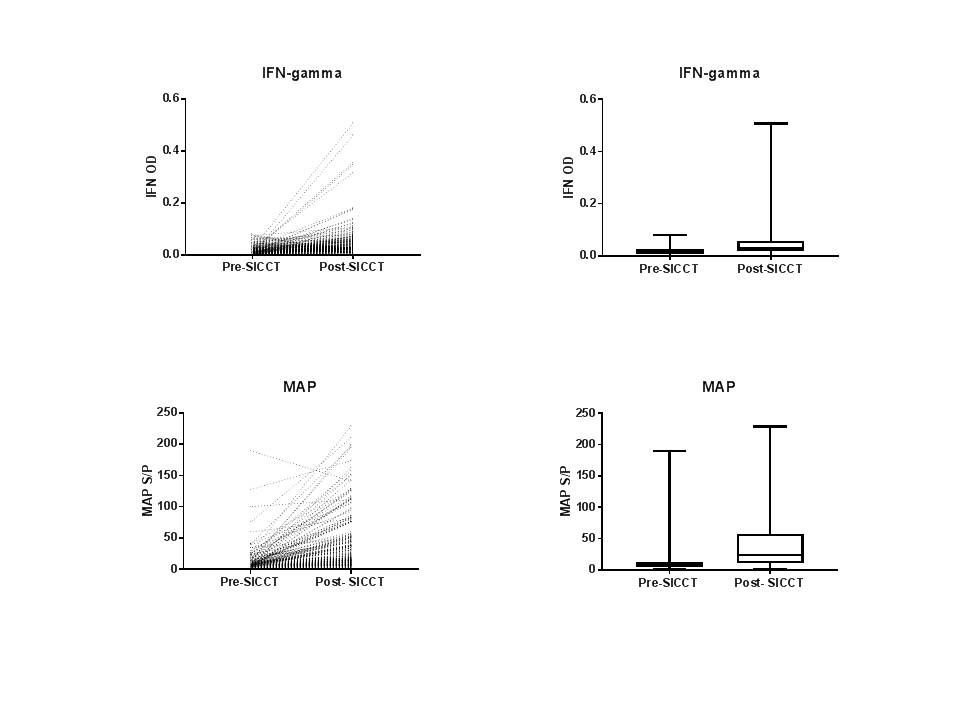

Supplement: Supplementary file 2 [file Image_1.JPEG]

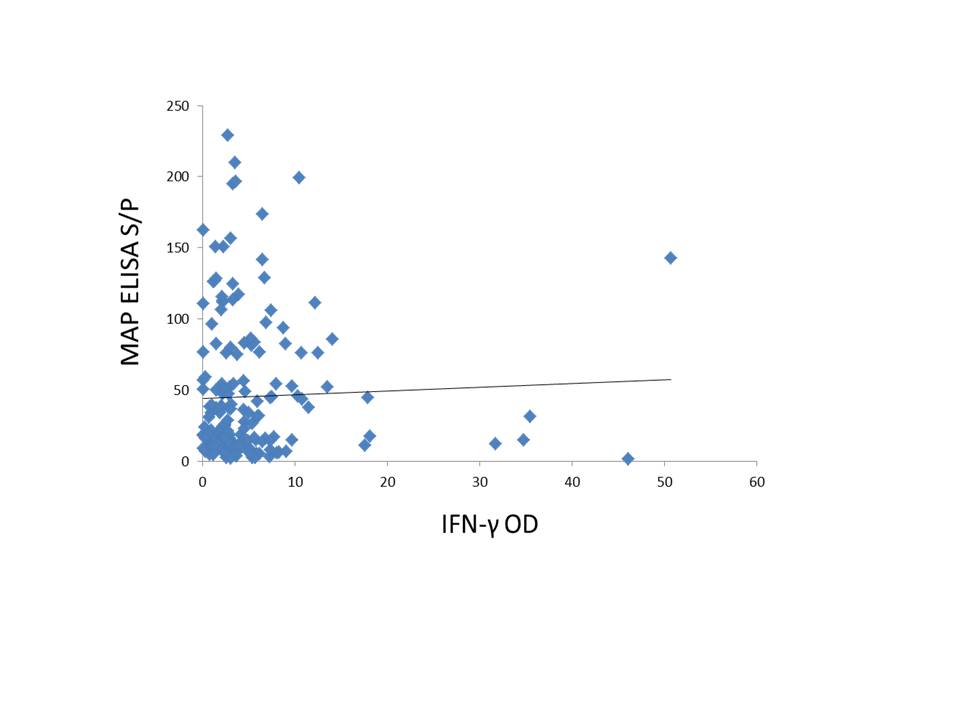

Supplement: Supplementary file 3 [file Image_2.JPEG]
